# RNA purification-free detection of SARS-CoV-2 using reverse transcription loop-mediated isothermal amplification (RT-LAMP)

**DOI:** 10.1186/s41182-021-00396-y

**Published:** 2022-01-04

**Authors:** Meng Yee Lai, Jeyanthi Suppiah, Ravindran Thayan, Ilyiana Ismail, Nur Izati Mustapa, Tuan Suhaila Tuan Soh, Afifah Haji Hassan, Kalaiarasu M. Peariasamy, Yee Leng Lee, Yee Ling Lau

**Affiliations:** 1 Department of Parasitology, Faculty of Medicine, University Malaya, 50603 Kuala Lumpur, Malaysia; 2 Virology Unit, Infectious Disease Research Centre, Institute for Medical Research, National Institutes of Health, Ministry of Health, Shah Alam, Selangor, Malaysia; 3 Department of Pathology, Hospital Sungai Buloh, Ministry of Health, Sungai Buloh, Selangor, Malaysia; 4 Institute for Clinical Research, National Institutes of Health, Ministry of Health, Shah Alam, Selangor, Malaysia; 5 Clinical Research Centre, Hospital Sungai Buloh, Ministry of Health, Sungai Buloh, Selangor, Malaysia

**Keywords:** COVID-19, Nasopharyngeal swab, SARS-CoV-2, RT-LAMP

## Abstract

**Background:**

Current diagnosis of SARS-CoV-2 infection relies on RNA purification prior to amplification. Typical extraction methods limit the processing speed and turnaround time for SARS-CoV-2 diagnostic testing.

**Methods:**

Here, we applied reverse transcription loop-mediated isothermal amplification directly onto human clinical swabs samples to amplify the RNA from SARS-CoV-2 swab samples after processing with chelating resin.

**Results:**

By testing our method on 64 samples, we managed to develop an RT-LAMP assay with 95.9% sensitivity (95% CI 86 to 99.5%) and 100% specificity (95% CI 78.2–100%).

**Conclusion:**

The entire process including sample processing can be completed in approximately 50 min. This method has promising potential to be applied as a fast, simple and inexpensive diagnostic tool for the detection of SARS-CoV-2.

**Supplementary Information:**

The online version contains supplementary material available at 10.1186/s41182-021-00396-y.

## Background

SARS-CoV-2 has caused a global COVID-19 pandemic disease. The number of reported COVID-19 cases worldwide has passed 180 million and deaths more than 4 million. To date, real time reverse transcription polymerase chain reaction (rRT-PCR) remains the gold standard for diagnosis. However, the turnaround time is long, requires sophisticated equipment and expensive probes which are not affordable in poor resource settings. There are several on-going efforts to minimize the PCR process and recent developments using loop-mediated isothermal amplification may offer a solution. The LAMP assay is highly specific as it involves 4 to 6 primers (forward inner primer, forward outer primer, reverse inner primer, and reverse outer primer) [1]. In addition, its short amplification time allows LAMP to be potentially developed as a point-of-care diagnostic tool.

In the current study, RNA was extracted by chelating resin and without further purification, the extracted RNA directly served as the template in RT-LAMP. Chelex-100 chelating resin has been used in DNA extraction from dried blood spots [2], mosquito [3], forensic materials [4], and plant materials [5, 6]. However, there are not many reports on RNA extraction using chelating resin. In this study, we demonstrated a simple RNA extraction from swab samples using chelating resin.

## Main text

### Methods

#### Sample collection

We used 49 rRT-PCR positive and 25 rRT-PCR negative nasopharyngeal and oropharyngeal swabs samples. The rRT-PCR threshold cycle value (*C*_T_-value) of these samples ranged from 13.95 to 38.85 (Additional file 1: Table S1). The swab samples which were collected in sterile vials containing viral transport medium (VTM) were preheated for 1 h at 65 °C. An aliquot of 250 μL of the swab samples were sent to laboratory in dry ice and used for evaluation of RT-LAMP assay. Ethical approvals were obtained from UMMC Medical Ethics Committee (202041-8418) and Medical Research Ethics Committee (MREC) Ministry of Health Malaysia (NMRR-20-2344-56994).

### RNA extraction

Total RNA extracted using chelating resin was according to the procedures described by Flynn et al. [7] with minor modification. A 30% Chelex-100 resin solution (BioRad Laboratories, US) was prepared in RNase-free water. The solution was mixed by 3 min vortex and kept in 4 °C until further use. A total of 15 μL of VTM was added to 22.5 μL of a 30% Chelex-100 resin solution, mixed by pipette up and down for 5 s, incubated at 95 °C, 10 min, cooled on ice for 2 min, followed by 30 s short spin. The supernatant was readily served as the template for amplification.

### RT-LAMP

The RT-LAMP assay was performed in a total of 25 μL reaction mixture consisting of 11 µL RNase free water, 2.5 µL of 10 × isothermal amplification buffer II, 1.5 µL of 100 mM MgSO_4_, 1.4 µL of 100 mM dNTPs, 1 µL of *Bacillus stearothermophilus* (*Bst*) 3.0 DNA polymerase (NEB, Ipswich, United States), 1 µL RNaseOUT recombinant ribonuclease inhibitor (Thermo Fisher Scientific, Massachusetts, United States), 0.3 µL of 10 mM hydroxynaphthol blue (HNB) (Sigma, St. Louis, United States), (NEB, Ipswich, United States), 3.8 µL of *N1* gene primers mix and 2.5 µL of template. The *N1* gene primers (Table 1) involved in this study were designed using the Primer-Explorer V4 software (Eiken Chemical Co., Ltd., Tokyo, Japan) based on SARS-CoV-2 nucleocapsid protein (GenBank accession no: MN988713.1, LC528233.1 and MT123293.1). The amplification was incubated in Loopamp Real-Time Turbidimeter LA 500 (Eiken Chemical Co., Ltd., Taito-ku, Japan) at 65 °C for 45 min. End point detection of RT-LAMP assay was performed by observing the colour changes of the end product. Positive LAMP reaction indicated sky blue and negative reaction remained in violet colour.

**Table 1:** Primers used in this study

Primer	Sequence (5' to 3')
FIP	TGGGGTCCATTATCAGACATTTTAGTTTTAGAGTATCATGACGTTCG
BIP	CGAAATGCACCCCGCATTACCCACTGCGTTCTCCATTC
FLP	TGTTCGTTTAGATGAAATC
BLP	TGGTGGACCCTCAGATTCAA
F3	GTTGTTCGTTCTATGAAGACT
B3	GACGTTGTTTTGATCGCG

Features: FIP, Forward inner primer; BIP, backward inner primer; FLP, forward loop primer; BLP, backward loop primer; F3, forward primer; B3, backward primer

### Analytical sensitivity and specificity, clinical sensitivity and specificity

Analytical sensitivity and specificity tests of *N1* gene had been tested and reported previously [8]. In this study, the clinical sensitivity and specificity of RT-LAMP were evaluated using 74 samples. Sensitivity was calculated based on the formula: (number of true positives)/(number of true positives + number of false negatives) and specificity was calculated as (number of true negatives)/(number of true negatives + number of false positives).

## Results and discussion

**Fig. 1:**
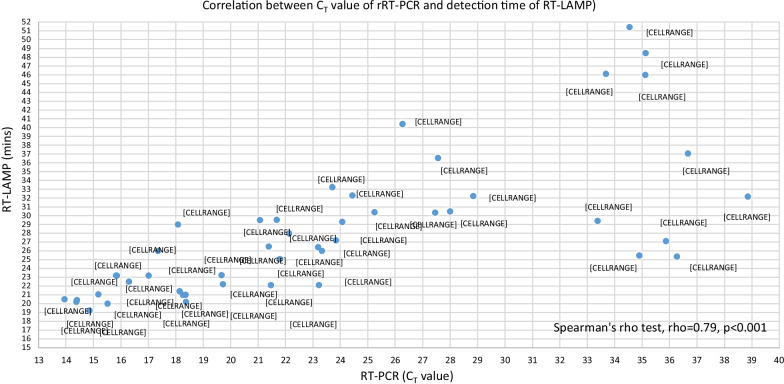
Correlation between *C*_T_ value of rRT-PCR and detection time of RT-LAMP. rRT-PCR, real time reverse transcription polymerase chain reaction; RT-LAMP, reverse transcriptase loop-mediated isothermal amplification; min, minutes; *C*_T_, cycle threshold

Based on our previous published report, analytical sensitivity and specificity tests for *N1* gene were 1 copy/µL RNA sensitive and 100% specific, respectively [8]. Out of 49 rRT-PCR positive samples, RT-LAMP detected 47 samples as positive and did not detect any of the rRT-PCR negative samples as positive. It was 95.9% sensitive (95% CI 86.0 to 99.5%) and 100% specific (95% CI 78.2–100%). Results showed that RT-LAMP did not detect two patients with low viral load (rRT-PCR C_T_ value of 35.19 and 36.06). The RNA in these samples maybe degraded during storage or shipping. There is correlation between C_T_ values of rRT-PCR and detection time of RT-LAMP. Figure 1 shows samples with high *C*_T_ value took a longer time to amplify by RT-LAMP. According to Spearman's rho test, there is strong correlation between *C*_T_ values of rRT-PCR and detection time of RT-LAMP. Both are statistically significant, rho = 0.79, *p* < 0.001. When *C*_T_ values of rRT-PCR increase, detection time of RT-LAMP also become longer. Nucleic acid extraction using chelating resin offers many advantages compared to conventional methods, such as spin/vacuum columns-based or paramagnetic beads-based extraction kits. These typical column- and paramagnetic beads-based methods are tedious as it involves multiple steps of processing and requires various consumables. A major drawback of these methods is the requirement of large quantities of biological samples for extraction [6]. However, using chelating resin for RNA extraction, only a minute volume of VTM samples (15 μL) was needed and the preparation of chelating resin was simple. The simple RNA preparation step improved diagnostic efficiency, cost and time. In addition, another benefit of using chelating resin for extraction was demonstrated by Walsh et al. [4] during their DNA extraction from forensic materials. They reported that DNA extraction from semen and blood stain samples using chelating resin was as sensitive if not more sensitive than proteinase K and phenol–chloroform extraction methods.

Coupled with the chelating resin extraction method, we achieved a promising result of 95.9% sensitivity for RT-LAMP. Flynn et al. [7] obtained 90% sensitivity during their optimization of RT-LAMP-based SARS-CoV-2 diagnostic protocol for saliva samples. Another study from Howson et al. [9] showed that overall sensitivity of direct RT-LAMP on saliva samples was approximately 83%.

Intriguingly, we managed to detect 10 samples with high *C*_T_ values (rRT-PCR *C*_T_ > 33.68–38.85) (Additional file 1: Table S1) in the current direct swab-to-RT-LAMP assay. This is a promising finding compared to other direct swab-to-RT-LAMP studies. Without further RNA extraction from nasopharyngeal swab samples, Lamb et al. [10] managed to detect 40% (*N* = 4/10) of SARS-CoV-2 (rRT-PCR *C*_T_ < 24) samples by RT-LAMP. In another study by Wei et al. [11], out of 13 clinical swab positive samples (rRT-PCR *C*_T_ < 31) tested, they only managed to detect 5 samples by RT-LAMP assay.

Various simplified preparation methods have been reported which can circumvent RNA extraction procedures due to the shortage of commercial kits in expanding diagnostic facilities of SARS-CoV-2. Fowler et al. [12] managed to obtain 67% sensitivity of RT-LAMP using VTM sample (1:20 dilution in water). Nie et al. [13] reported a direct RT-LAMP by heating nasopharyngeal swab samples at 95 °C for 30 s and cooling on ice for 2 min. In addition, Dao Thi et al. [14] managed to obtain 86% sensitivity RT-LAMP using swab samples either without any treatment or after heat treatment for 5 min at 95 °C during their development of colorimetric RT-LAMP assay. As for the high sensitivity of direct RT-LAMP, Yoshikawa et al. [15] also reported 1.43 × 10^3^ copies of RNA by direct heating the swab samples at 95 °C for 10 min. However, we were not able to replicate these methods after several trials. The failure may be due to the presence of inhibitors, such as glucose in VTM.

Apart from real time turbidity detection of RT-LAMP results, the results can also be examined by observing the colour changes of the end product. We included HNB colorimetric dye in the LAMP assay during the preparation of the master mix. Carryover contamination can be eliminated as the dye was not added into the reaction tube upon completion of the amplification. Typical approach to confirm the LAMP results was performed by running the end products via gel electrophoresis. Unfortunately, running end products by gel electrophoresis may contribute to the issue of carryover contamination.

LoopAmp Real-Time Turbidimeter LA 500 used in this study can be upgraded with six control units, at which, 96 RT-LAMP tests can be performed in 1 h. For high-throughput testing, multiple instruments can be employed.

Even though SARS-CoV-2 antigen-detection tests that offer results in 15 min and as they become more widely available as at-home, self-administered and over-the-counter tests at pharmacy stores, our RT-LAMP assay still can be considered as an alternative for SARS-CoV-2 testing especially in resource limited areas. Most of these tests show lower sensitivity as compared to our previous published RT-LAMP assay (100% sensitivity) [16]. Meanwhile, Harmon et al. [17] reported 78.9% sensitivity using an at-home direct antigen rapid test kit. Another study reported by Shrestha et al. (2020) also revealed that low sensitivity of lateral flow antigen test kits for COVID-19 testing with 85% sensitivity [18]. Moreover, the developed RT-LAMP here costs only USD$1.95/reaction as compared to antigen detection test (USD$4.30).

## Conclusions

We have successfully developed an optimized direct swab-to-RT-LAMP assay without RNA purification. This method is sufficiently sensitive in detecting SARS-CoV-2 directly from clinical nasopharyngeal swab samples in VTM. Given these results and coupled with RT-LAMP assay, this methodology can facilitate the expansion of SARS-CoV-2 diagnostic testing especially in resource limited areas.

## Supplementary Information

**Additional file 1. Table S1**. Result of rRT-PCR (CT –value) and RT-LAMP (mins).

## Data Availability

The data set for this study is available from the corresponding author and Hospital Sungai Buloh on a reasonable request. Data without names and identifiers will be made available after approval from the corresponding author and Hospital Sungai Buloh.

## Abbreviations

RNA: Ribonucleic acid
SARS-CoV-2: Severe acute respiratory syndrome coronavirus 2
RT-LAMP: Reverse transcription loop-mediated isothermal amplification (RT-LAMP)
COVID-19: Coronavirus disease
rRT-PCR: Real time reverse transcription polymerase chain reaction (rRT-PCR)
*C*_T_: Threshold cycle value
VTM: Viral transport medium
UMMC: University Malaya Medical Center
MREC: Medical Research Ethics Committee
dNTPs: Deoxynucleoside triphosphate
HNB: Hydroxynaphthol blue
*Bst*: *Bacillus stearothermophilus*
NEB: New England Biolabs
Mins: Minutes
CI: Confidence intervals
