# Correction: RNA purification-free detection of SARS-CoV-2 using reverse transcription loop-mediated isothermal amplification (RT-LAMP)

**DOI:** 10.1186/s41182-023-00555-3

**Published:** 2023-11-22

**Authors:** Meng Yee Lai, Jeyanthi Suppiah, Ravindran Thayan, Ilyiana Ismail, Nur Izati Mustapa, Tuan Suhaila Tuan Soh, Affah Haji Hassan, Kalaiarasu M. Peariasamy, Yee Leng Lee, Yee Ling Lau

**Affiliations:** 1 Department of Parasitology, Faculty of Medicine, University Malaya, 50603 Kuala Lumpur, Malaysia; 2 Virology Unit, Infectious Disease Research Centre, Institute for Medical Research, National Institutes of Health, Ministry of Health, Shah Alam, Selangor Malaysia; 3 Department of Pathology, Hospital Sungai Buloh, Ministry of Health, Sungai Buloh, Selangor Malaysia; 4 Institute for Clinical Research, National Institutes of Health, Ministry of Health, Shah Alam, Selangor Malaysia; 5 Clinical Research Centre, Hospital Sungai Buloh, Ministry of Health, Sungai Buloh, Selangor Malaysia

**Correction: Tropical Medicine and Health (2022) 50:2** **https://doi.org/10.1186/s41182-021-00396-y**

Following publication of the original article [1], the authors reported that the Funding section needed to be updated.

The original version was:

**Funding:**

This study was supported by Prototype Research Grant Scheme (PRGS), PR001-2020B (PRGS/2/2020/SKK09/UM/02/1) from the Ministry of Education, Malaysia.

The updated version is:

**Funding:**

This study was supported by the Prototype Research Grant Scheme (PRGS/2/2020/SKK09/UM/02/1) from the Ministry of Higher Education, Malaysia.

The original article [1] has been corrected.
