# Supplementary material for: RNA purification-free detection of SARS-CoV-2 using reverse transcription loop-mediated isothermal amplification (RT-LAMP)
Source: Trop Med Health. 2022 Jan 4;50:2. doi: 10.1186/s41182-021-00396-y (PMC8723997; doi:10.1186/s41182-021-00396-y)
Supplement: Supplementary file 1 — Additional file 1. Table S1. Result of rRT-PCR (CT –value) and RT-LAMP (mins). [file 41182_2021_396_MOESM1_ESM.docx]

**Table S1**

| **Sample** | **rRT-PCR (**C_T_ –value) | **RT-LAMP (mins)** |
| --- | --- | --- |
| N8585 | 21.80 | 25.06 |
| N8586 | 19.72 | 22.20 |
| N8588 | 18.08 | 29.00 |
| N8589 | 18.36 | 21.01 |
| N8598 | 17.35 | 26.00 |
| N8669 | 25.25 | 30.40 |
| N8759 | 23.84 | 27.20 |
| N8829 | 15.51 | 20.00 |
| N8888 | 27.45 | 30.36 |
| 42889 | 18.37 | 20.20 |
| 42903 | 16.29 | 22.50 |
| 45107 | 14.38 | 20.20 |
| 355250 | 13.95 | 20.50 |
| 51716 | 22.13 | 28.00 |
| 43503 | 21.07 | 29.50 |
| 46759 | 15.85 | 23.20 |
| 25306 | 17.01 | 23.20 |
| 39298 | 21.47 | 22.10 |
| 65760 | 14.86 | 19.20 |
| 343120 | 15.82 | 23.20 |
| 342150 | 18.14 | 21.42 |
| 342149 | 23.33 | 26.00 |
| 342649 | 19.67 | 23.24 |
| 342652 | 21.68 | 29.54 |
| 342655 | 21.39 | 26.50 |
| 342660 | 24.44 | 32.30 |
| 342678 | 24.07 | 29.30 |
| 341923 | 15.18 | 21.06 |
| 342328 | 14.4 | 20.4 |
| N10556 | 23.19 | 26.42 |
| N10609 | 28.84 | 32.24 |
| N10606 | 23.71 | 33.24 |
| N10596 | 27.56 | 36.54 |
| N10540 | 18.26 | 21.00 |
| N10229 | 23.22 | 22.12 |
| N10597 | 36.27 | 25.36 |
| N10348 | 35.87 | 27.12 |
| N10467 | 36.67 | 37.06 |
| N10184 | 38.85 | 32.18 |
| N10440A | 33.38 | 29.42 |
| N10376A | 34.90 | 25.48 |
| N10463 | 35.19 | Negative |
| N10241 | 36.06 | Negative |
| 134400 | 35.13 | 48.48 |
| 135983 | 34.54 | 51.42 |
| 136346 | 35.12 | 46.00 |
| 135466 | 26.27 | 40.42 |
| 135475 | 28.00 | 30.50 |
| 136304 | 33.68 | 46.12 |
| N10454 | Negative | Negative |
| N10455 | Negative | Negative |
| N10472 | Negative | Negative |
| N10481 | Negative | Negative |
| N10479 | Negative | Negative |
| N10422 | Negative | Negative |
| N10435 | Negative | Negative |
| N10441 | Negative | Negative |
| N10458 | Negative | Negative |
| N10459 | Negative | Negative |
| N8795 | Negative | Negative |
| 76247 | Negative | Negative |
| 76248 | Negative | Negative |
| 342136 | Negative | Negative |
| 342137 | Negative | Negative |
| 136646 | Negative | Negative |
| 136647 | Negative | Negative |
| 136648 | Negative | Negative |
| 136649 | Negative | Negative |
| 136650 | Negative | Negative |
| 136651 | Negative | Negative |
| 136652 | Negative | Negative |
| 136653 | Negative | Negative |
| 136654 | Negative | Negative |
| 136657 | Negative | Negative |

C_T_-values: The C_T_ (cycle threshold) is defined as the number of cycles required for the fluorescent signal to cross the threshold.
